# Tianshanbeilu and the Isotopic Millet Road: reviewing the late Neolithic/Bronze Age radiation of human millet consumption from north China to Europe

**DOI:** 10.1093/nsr/nwx015

**Published:** 2017-02-24

**Authors:** Tingting Wang, Dong Wei, Xien Chang, Zhiyong Yu, Xinyu Zhang, Changsui Wang, Yaowu Hu, Benjamin T Fuller

**Affiliations:** 1 Key Laboratory of Vertebrate Evolution and Human Origins of Chinese Academy of Sciences, Institute of Vertebrate Palaeontology and Palaeoanthropology, Chinese Academy of Sciences, Beijing 100044, China; 2 Department of Archaeology and Anthropology, University of Chinese Academy of Sciences, Beijing 100049, China; 3 Department of Anthropology, School of Sociology and Anthropology, Sun Yat-sen University, Guangzhou 510275, China; 4 Research Center for Chinese Frontier Archaeology, Jilin University, Changchun 130012, China; 5 Institute of Archaeology and Cultural Relics of Xinjiang Uyghur Autonomous Region, Urumqi 830000, China

**Keywords:** Inner Asian Mountain Corridor, Silk Road, Xinjiang, Old World crop globalization, Shang Dynasty

## Abstract

The westward expansion of human millet consumption from north China has important implications for understanding early interactions between the East and West. However, few studies have focused on the Xinjiang Uyghur Autonomous Region, the vast geographical area directly linking the ancient cultures of the Eurasian Steppe and the Gansu Corridor of China. In this study, we present the largest isotopic investigation of Bronze Age China (*n* = 110) on material from the key site of Tianshanbeilu, in eastern Xinjiang. The large range of δ^13^C values (–17.6‰ to –7.2‰; –15.5 ± 1.2‰) provides direct evidence of unique dietary diversity and consumption of significant C_4_ resources (millets). The high δ^15^N results (10.3‰ to 16.7‰; 14.7 ± 0.8‰) likely reflect sheep/goat and wild game consumption and the arid climate of the Taklamakan Desert. Radiocarbon dates from four individuals indicate Tianshanbeilu was in use between 1940 and 1215 cal bc. The Tianshanbeilu results are then analysed with respect to 52 Bronze Age sites from across Eurasia, to investigate the spread and chronology of significant human millet consumption and human migration. This isotopic survey finds novel evidence that the second millennium bc was a dynamic period, with significant dietary interconnectivity occurring between north China, Central Asia and Siberia. Further, we argue that this ‘Isotopic Millet Road’ extended all the way to the Mediterranean and Central Europe, and conclude that these C_4_ dietary signatures of millet consumption reflect early links (migration and/or resource transfer) between the Bronze Age inhabitants of modern-day China and Europe.

## INTRODUCTION

**Figure 1:**
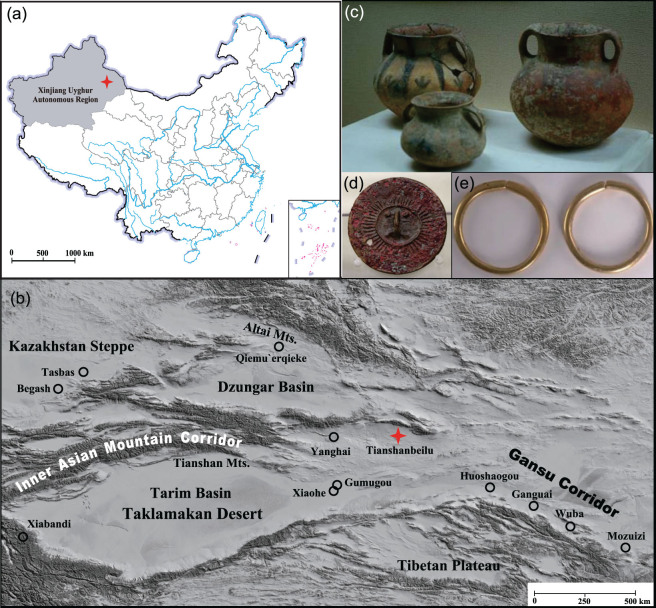
(a) and (b) Maps showing the location of the Tianshanbeilu cemetery site (✦) in the Xinjiang Uyghur Autonomous Region of China; (c) Color-painted potteries (http://www.bangbenw.com/yx/tpk/2010-08/25/content_903355.htm); (d) Bronze mirror with image of a human face (http://www.cchmi.com/tabid/702/InfoID/11395/Default.aspx); (e) Gold earrings from tomb M325 (individual not studied) excavated at Tianshanbeilu (http://www.xjbs.com.cn/news/2015-04/01/cms1756084article.shtml).

Before the Silk Road introduced the luxuries and technologies of China to Central Asia/Europe, a collection of regional and non-formalized long-distance networks of trade and communication, maintained by nomadic pastoralists, connected the East and West (e.g. Trans-Eurasian exchange, Proto-Silk Road, Inner Asian Mountain Corridor, etc.) [1–8]. Located at the geographical confluence of eastern and western cultures, the modern-day Xinjiang Uyghur Autonomous Region (Fig. 1a and b) served as a bridge between the ancient tribes of the Eurasian Steppe and the peoples of the Gansu Corridor of modern-day China [9–13]. Recently, genetic studies found that the Bronze Age of the Eurasian continent was a dynamic period that witnessed frequent human interactions and migrations [14–17]. The eastward movement of Indo-European populations into China resulted in the introduction of numerous south-west Asian crops and domesticated animals [18–21] as well as technological advances (e.g. metalworking, horse riding, wheeled vehicles) and changes in social structure [1,7,22,23]. Archaeological evidence indicates that by ~2000 bc, Indo-Europeans were migrating to the Tarim Basin from Central Asia [1,5,24] and components of the Afanasyevo (3700–2500 bc), Okunevo (2500–1900 bc) and the Andronovo (1900–1500 bc) cultural complexes are found incorporated into some of the earliest known archaeological sites of Xinjiang such as Qiemu’erqieke (a.k.a. Ke’ermuqi) [25–27], Xiaohe [14,28,29] and Gumugou [30,31].

However, this exchange was not unidirectional and crops, technologies and peoples also journeyed westward from the cultural centers of the Yellow River Valleys of China [12,19,32–35]. In terms of cultigens, foxtail millet (*Setaria italica*) and common millet (*Panicum miliaceum*) were vital resources for the ancient inhabitants of north China with a long history of cultivation and consumption [36–41]. At some point between the third to second millennium bc, but possibly earlier, millet was transported to the West and eventually adopted by populations outside of China [42]. At present, the earliest undisputed evidence for millet beyond the borders of modern China is the direct radiocarbon dates of millet grains (2460–2150 cal bc) at the site of Begash in southern Kazakhstan [12,43]—a steppe area linked to the Gansu Corridor by the Tianshan Mountains of China (Fig. 1b). Archaeobotanical studies have also discovered millet remains at numerous other Bronze Age archaeological sites along the Tianshan Mountains, and its nearby mountain ranges [35], which together form the Inner Asian Mountain Corridor [6]. This Inner Asian Mountain Corridor is believed to be a key artery for early East–West interactions [6,10,12] and was possibly bound together by the common cultures of the Srubnaya (1800–1200 bc) and Andronovo (1900–1500 bc) of the western and eastern Eurasian Steppes, respectively [23].

While archaeobotanical studies yield invaluable information about the presence of a particular cultigen at an archaeological site, they do not provide direct evidence that these crops were consumed in significant amounts by a population [13,34,44]. However, carbon (δ^13^C) and nitrogen (δ^15^N) stable isotope ratio analysis provides a direct method to reconstruct human and animal dietary patterns [45–47]. In particular, δ^13^C signatures are well suited as natural biomarkers of millet consumption in China and Eurasia due to the large isotopic differences between important C_3_ (e.g. wheat (*Triticum aestivum*), barley (*Hordeum vulgare*)) and C_4_ cultigens (e.g. foxtail and common millet). For example, C_3_ plants select against ^13^C when fixing CO_2_, and thus have more negative δ^13^C values (–30‰ to –22‰) than C_4_ plants (–16‰ to –10‰), with mean values of –26.5‰ and –12.5‰, respectively [48–51]. These different plant δ^13^C values are then incorporated into a consumer's body tissues with known corresponding offsets or fractionation factors [52,53]. To date, stable isotope studies in archaeology are most often carried out on bone collagen, and +5‰ is commonly suggested as the offset between bone collagen and diet for mammals [54–59]. For studies of bone collagen, it is also estimated that ~20% of the dietary protein must originate from C_4_ sources in order for the isotopic signatures to be distinguishable from a predominately C_3_ diet. Previous research has defined a δ^13^C cut-off value of approximately –18‰ as the difference between predominately C_3_ and mixed C_3_/C_4_ diets and a value of –12‰ as the approximate boundary between mixed C_3_/C_4_ diets and predominately C_4_ diets [50,60,61].

Here, results of the largest isotopic study of Bronze Age China (*n* = 110), as well as radiocarbon dates from four individuals, are presented to investigate human diet and age of habitation at the Tianshanbeilu (TB) cemetery site in the Xinjiang Uyghur Autonomous Region of China. TB is regarded as one of the most important archaeological sites in eastern Xinjiang, and displays a vast wealth of archaeological, anthropological and genetic evidence for significant links between the cultures of the Gansu Corridor and the Eurasian Steppe (Fig. 1a–e, Archaeological Background). Over 3000 artifacts (bronze, pottery, bone, stone, gold, silver, cowries, etc.) were discovered from the approximately 700 tomb burials [62] and the large regional diversity of these items indicate some of the earliest and most dynamic cultural interactions between the East and the West in China [24]. Only limited isotopic research has been published for Bronze Age sites from the Xinjiang region [63–65], including a pilot isotopic study of 10 individuals from TB [66] that revealed mixed C_3_/C_4_ diets. In addition to our new data, we conduct a detailed review of 52 Bronze Age archaeological sites, comprising >1000 individuals, from across Eurasia. Here, our aim is to use these human δ^13^C and δ^15^N results as natural isotopic tracers to construct the ‘Isotopic Millet Road’ (note this could equally be called the ‘Millet Road’ if focused primarily on archaeobotanical remains), which can be used to investigate the westward radiation of millet consumption from the Yellow River Valleys. These results are plotted to visualize sites from the second millennium bc where individuals who consumed significant amounts of millet have been recovered and to identify the many possible routes of interconnectivity between the Yellow River Valleys of north China, Central Asia, Siberia and Europe.

### Archaeological background

TB, also known as Linya, is located near the modern city of Hami in the Xinjiang Uyghur Autonomous Region of China in the eastern portion of the Inner Asian Mountain Corridor (Fig. 1a and b). TB is the largest Bronze Age cemetery site found in eastern Xinjiang to date [24,67]. Incorporating characteristics of the cultures from the Eurasian Steppes and the Gansu Corridor, the grave goods of the TB cemetery are remarkably diverse and indicate some of the earliest and most dynamic cultural interactions between the East and the West in China [24].

Large quantities of pottery discovered at TB were assigned to two different unique typologies (Fig. 1c). Group one has similar morphology, materials, and painted patterns with the pottery of the post-Machang and Siba cultures (~2000–1500 bc) from the Gansu Corridor [68]. Group two resembles pottery of the western Qiemu’erqieke (a.k.a. Ke`ermuqi) culture (~2000–1500 bc) from the Altai Mountains [4,25,26,69] and/or the Andronovo culture from the Eurasian Steppes [2,3,69]. Moreover, burial styles and bronze artifacts at TB display characteristics of the Eurasian Steppe cultures (Fig. 1d). The discovery of bronze drills and curved-backed bronze knives suggest these originated from the Okunevo culture (2500–1900 bc) of south Siberia, while the short bronze sword, sun-dried mud brick and the solid wooden wheel were possibly from the Sintashta-Petrovka culture (2100–1800 bc) of the South Ural region [4]. As presented in Fig. 1e, some of the earliest gold artifacts in Xinjiang, a pair of earrings, were also found in tomb M325 (individual not studied) and these are representative of the type recovered from the steppe areas of Eurasia. Physical anthropology and ancient DNA analysis support the genetic mixing of peoples from both the East and West at TB [67,69–71]. Examination of the morphological indexes from the cranium of 32 TB individuals found the population had some physical characteristics of both Mongolians and Europeans [62,67,70]. Mitochondrial DNA and Y-STR analysis was also conducted on 29 TB individuals [69] and 10 different haplogroups were detected, among which eight originate from East Eurasia, while the other two haplogroups (U and W) are considered West Eurasian lineages and are widely distributed in Siberia, Europe as well as in central and south-west Asia in the Neolithic.

**Figure 2:**
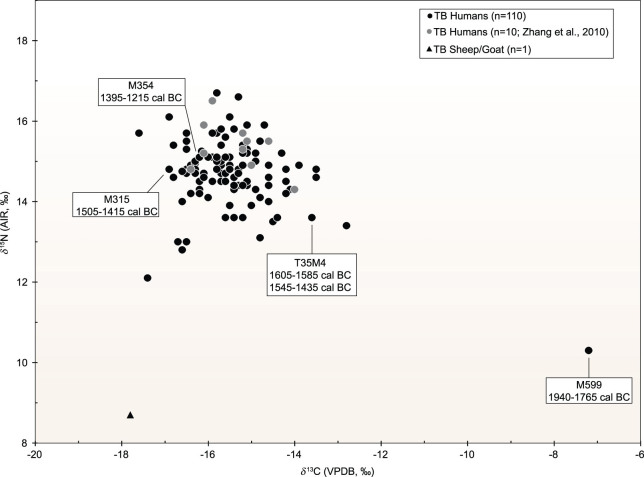
Human and sheep/goat δ^13^C and δ^15^N values from Tianshanbeilu. Ages of four individuals directly radiocarbon dated are also shown. See Table S1 (available as Supplementary Data at *NSR* online) for additional details.

## MATERIALS AND METHODS

Collagen was isolated from 110 adult humans and one sheep/goat at the Key Laboratory of Vertebrate Evolution and Human Origins of Chinese Academy of Sciences, Institute of Vertebrate Palaeontology and Palaeoanthropology, Chinese Academy of Sciences using the protocol outlined in Richards and Hedges [72]. Collagen was measured at the Environmental Stable Isotope Laboratory (ESIL), Institute of Environment and Sustainable Development of Agriculture, Chinese Academy of Agricultural Sciences and at the Archaeological Stable Isotope Laboratory (ASIL), the Department of Archaeology and Anthropology at the University of the Chinese Academy of Sciences. The mass spectrometers were both IsoPrime 100 IRMS coupled with Elementar Vario. The isotopic results analyse the ratio of the heavier isotope to the lighter isotope (^13^C/^12^C or ^15^N/^14^N) and are reported as ‘δ’ in parts per 1000 or ‘per mil’ (‰) relative to the internationally defined standards for carbon (Vienna Pee Dee Belemnite, VPDB) and nitrogen (Ambient Inhalable Reservoir, AIR). The standards were Sulfanilamide, IAEA-600, IEAE-N-1, IAEA-N-2, IAEA-CH-6, USGS-24, USGS 40 and USGS 41 and, for every 10 samples, a collagen lab standard (δ^13^C value of 14.7 ± 0.2‰ and δ^15^N value of 6.9 ± 0.2‰) was also inserted in the run for isotopic calibration. The measurement errors were less than ±0.2‰ for both δ^13^C and δ^15^N values.

## RESULTS

The sheep/goat and adult human sample information and isotopic results are presented in Table S1 (available as Supplementary Data at *NSR* online) and Fig. 2. All specimens had excellent preservation and produced good-quality collagen with C:N between 2.9 and 3.6 [73]. The sheep/goat has a δ^13^C value of –17.8‰ and a δ^15^N value of 8.7‰, which reflects a mainly C_3_ diet. The adult human δ^13^C results show a wide range of values (~10‰), from –17.6‰ to –7.2‰ (mean ± SD; –15.5 ± 1.2‰). In addition, the δ^15^N results are high and have a large range of values (~6‰) from 10.3‰ to 16.7‰ (mean ± SD; 14.7 ± 0.8‰). Radiocarbon dates on bone collagen from four individuals show that TB was in use from at least 1940 to 1215 cal bc.

**Figure 3:**
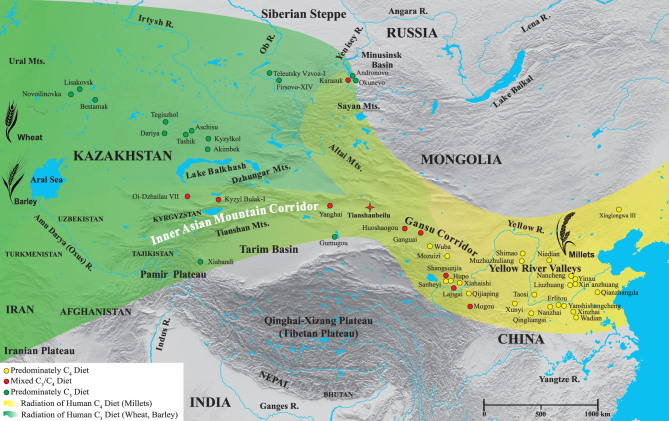
Map showing the spread of human millet consumption into Xinjiang and Central Asia along the Gansu and Inner Asian Mountain Corridors from the Yellow River Valleys during the second millennium bc.

## DISCUSSION

### Diet and radiocarbon dating at TB

In the northern latitudes of Eurasia, the two Asian millets (foxtail and common) are the only indigenous C_4_ crops likely to have been consumed in large quantities by ancient populations, and thus are responsible for the ^13^C-enriched isotopic signatures in humans and animals [9,34,74,75]. This makes δ^13^C values particularly sensitive markers with which to construct the Isotopic Millet Road, which can be used to detect the consumption and spread of millets from north China across the Eurasian Steppes and into Europe [34,39,47,76–80]. In this context, the isotopic results show that the TB humans consumed a significant amount of millets in their diets. Due to a lack of faunal isotope data, it is difficult to determine the specific type of animal protein consumed. However, the large number of sheep/goat remains excavated at TB [81] can be used to suggest these were a significant source of protein. In addition, wild game and freshwater fish were certainly eaten, as these foods were important components to human diets in Central Asia [9,82,83]. It is also noted that the high δ^15^N values of the TB humans (14.7‰) were also likely influenced by the arid environmental conditions of the Xinjiang region (see below).

Based on archaeological evidence, TB was occupied between 2000 and 1300 bc [24,67,70]. However, to determine a more precise chronology for the dietary patterns, four individuals with diverse isotopic values were directly radiocarbon dated (Table S1 (available as Supplementary Data at *NSR* online) and Fig. 2). The range of radiocarbon ages (1940–1215 cal bc) agrees with the archaeological evidence [84]. Interestingly, individual M599, with an exclusive C_4_ diet (millet), was also the oldest (1940–1765 cal bc). As this dietary pattern is characteristic of populations from the Gansu Corridor and the Yellow River Valleys, this discovery is possible evidence that there was an early movement of people from these regions to the TB area, which mirrors the archaeological and genetic evidence of extensive East–West interactions at this site (Archaeological Background).

**Figure 4:**
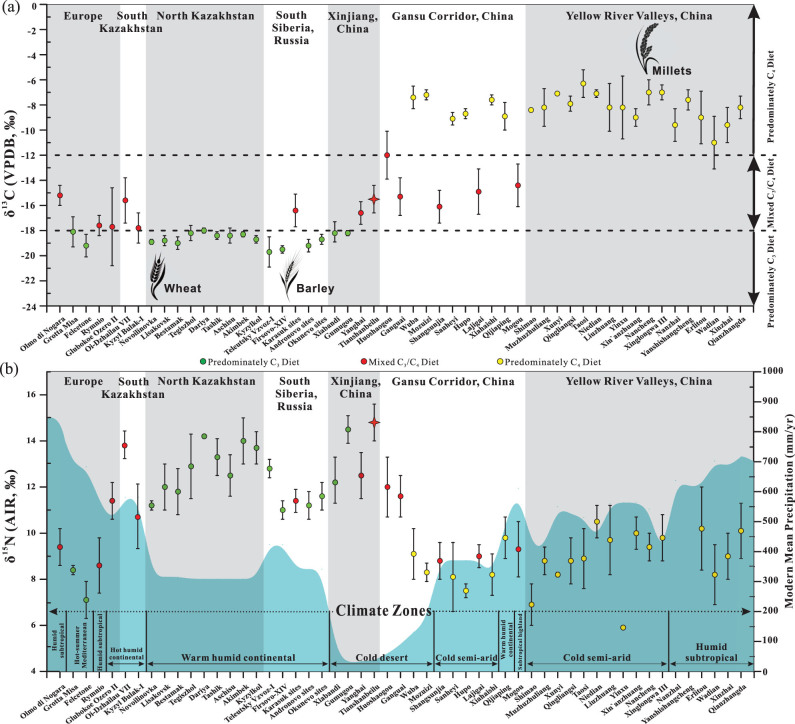
Mean ± SD human δ^13^C (a) and δ^15^N (b) results from all Bronze Age sites surveyed along the Isotopic Millet Road. For detailed results and references, see Tables S2 and S3 (available as Supplementary Data at *NSR* online). Elevated δ^15^N values at western Gansu Corridor and Xinjiang sites are likely influenced by the increased aridity of the Taklamakan Desert and modern mean annual precipitation and general climate zones are shown in (b). Note: measurements > –12‰ reflect a predominately C_4_ diet, values between –12‰ and –18‰ indicate a mixed C_3_/C_4_ diet and values < –18‰ reflect a predominately C_3_ diet in China [39,76].

**Figure 5:**
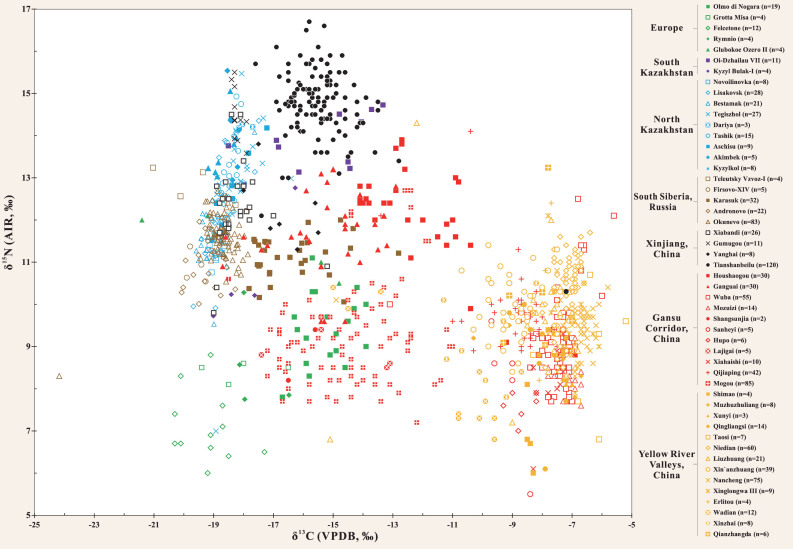
Scatter plotting the isotopic results of >1000 individuals from 53 Bronze Age sites from across Eurasia.

**Figure 6:**
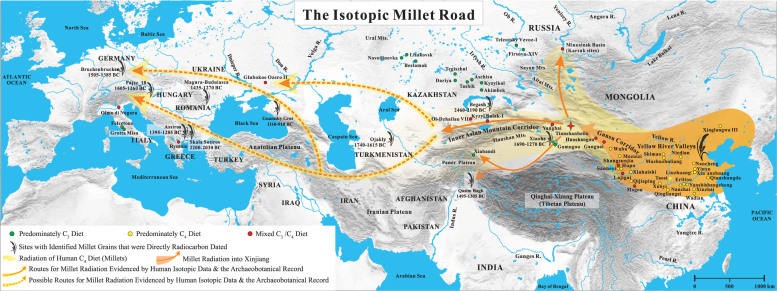
Map showing the Isotopic Millet Road for the radiation of human millet consumption from the Yellow River Valleys of China to Europe.

### The Isotopic Millet Road

Here, the stable isotope results of human remains (from studies with at least three individuals) from north China and bordering regions, Central Asia and Europe are examined (Table S2 (available as Supplementary Data at *NSR* online) and Figs 3–6). Our focus is mainly the second millennium bc, but, as many sites have no radiocarbon dates directly paired with isotopic results and since many sites also have large ranges of occupation, some late-third-millennium bc sites are also included. However, sites which mainly date to the first millennium bc or later were excluded. In addition, the δ^15^N results are plotted in relation to the modern mean annual precipitation (mm/yr) and the general climate zones of the individual sites (Table S3 (available as Supplementary Data at *NSR* online) and Fig. 4b) to visualize potential isotopic correlations with environmental conditions, such as aridity [85,86]. We acknowledge that the climate/precipitation changed considerably in the last 4000 years [87], but believe that these modern comparisons still provide valuable information with which to understand the human δ^15^N values, in addition to dietary intake. Here, the objectives are to examine the significance of the TB isotopic results in relation to other archaeological sites from the same general period. This permits the visualization of where and when there was significant dietary contact and exchange between the cultures of the Gansu Corridor/Yellow River Valleys and the Eurasian Steppe by using the δ^13^C signatures as proxies for human millet consumption.

### Yellow River Valleys and the Gansu Corridor

The humans from the Yellow River Valleys have significantly elevated δ^13^C results (~–6‰ to –11‰; Fig. 4a). This illustrates how all diets at this period across north China were heavily dominated by millets and were relatively homogenous at the population level, although large differences in isotope values between individuals existed [40,44,75,88–101]. The δ^15^N values are between ~6‰ and 10‰, and again there is significant variation, which accounts for the high standard deviations (Fig. 4b). The modern climate is variable at these sites, and the modern mean precipitation ranges between ~350 and 700 mm/yr. In the Gansu Corridor, numerous populations also have ^13^C-enriched results similar to the Yellow River Valleys, but exceptions exist, with mixed C_3_/C_4_ diets occurring at Mogou, Lajigai, Shangsunjia, Ganguai and Huoshaogou. The δ^15^N values range between ~8‰ and 10‰, except for the far western populations of Ganguai and Huoshaogou, which show elevated results (~11‰ to 12‰). In addition, traversing from east to west along the Gansu Corridor, the modern mean precipitation steadily declines and reaches a value of 58 mm/yr near Huoshaogou.

As can be seen in Figs 5 and S1 (available as Supplementary Data at *NSR* online), the Yellow River Valley sites display a large isotopic range and have significant overlap with the Gansu Corridor sites: Qijiaping, Xiahaishi, Mozuizi and Wuba, but are different compared to Mogou, Lajigai, Shangsunjia, Ganguai and Huoshaogou. Further, there are many significant outliers suggesting the possibility that interconnectivity in terms of human migration was likely occurring between these regions. For example, at the most eastern site of Mogou, the δ^13^C results indicate that there was less millet consumption by the population. This site has a long period of occupation (1750–1100 cal bc) and the radiocarbon dates and mixed C_3_/C_4_ isotopic results are similar to TB [44]. The three outliers from Wadian plot within the main cluster of individuals from Mogou, while two individuals from Mogou have similar results to Xinzhai and the three outliers from Qijiaping (Fig. 5). At Liuzhuang, a significant outlier possibly originated from Huoshaogou or TB and, at Nancheng, a single individual is observed to plot near the Mogou group. The Wuba site also has an individual that plots with the Mogou data. Further, Mogou has seven individuals with elevated δ^13^C and δ^15^N values that directly cluster with data from Huoshaogou and a single individual outlier that plots with individuals from the sites of the Minusinsk Basin of Russia (Fig. 5). This wide diversity of isotopic results, in conjunction with the finding of starch grains of wheat, barley and millet in the dental calculus of three skeletons from Mogou [102], suggests that there was significant East–West contact occurring at the far eastern end of the Gansu Corridor during the Bronze Age [44]. Further, some individuals were buried with Andronovo-style bronze earrings [103], indicating that at least resource transfer and possible migration of individuals from the Eurasian Steppe (e.g. Sintashta-Petrovka culture) was occurring at Mogou.

In contrast, Huoshaogou is the most western Bronze Age site of the Gansu Corridor and is located at the edge of the Taklamakan Desert. It shows some isotopic overlap with Mogou (~800 km to the south-east), as seven individuals found at Mogou plot with the Huoshaogou data. In addition, three individuals found at Huoshaogou have carbon and nitrogen isotopic similarities to individuals from the Yellow River Valley sites, suggesting that there was communication and migration between these locations (Fig. 5) [44]. Huoshaogou offers compelling evidence (in the form of radiocarbon dates directly paired with human δ^13^C values and wheat grains) for not only East–West contact, but also when this human interaction was initiated. Liu *et al.* [44] found that, before ~2000 cal bc, the diet at Huoshaogou was nearly exclusively based on millets, as seen at sites in the Yellow River Valleys. However, by ~1800 cal bc, the human δ^13^C signatures reflecting C_4_ plant consumption were diminished and, by ~1600–1300 cal bc, individuals consumed a mixed C_3_/C_4_ diet, which is in agreement with the TB results. Further, these isotopic findings at Huoshaogou are also supported by the direct radiocarbon dates of wheat grains, with the results demonstrating that wheat was present between 1880 and 1620 cal bc [104]. This collective evidence offers strong support that the cultures of what is now modern-day Gansu Province and the Eurasian Steppe (e.g. Sintashta-Petrovka, Andronovo) were in direct contact by at least 1800 bc (which coincides with the dates of the earliest Tarim mummies [1,30]). That these interactions intensified in terms of diet by approximately 1600 bc is relevant to Chinese Bronze Age archaeology, as this time coincides with the establishment and rise of the Shang Dynasty (~1600–1046 bc [1,36]).

### Xinjiang

In Xinjiang, this trend of decreasing δ^13^C values with westerly longitude continues and there is no longer evidence that the populations were consuming diets predominately based on millet (Fig. 4a). Interestingly, the δ^15^N values are elevated (~12‰ to 15‰; Fig. 4b), and all sites are located in and around the Taklamakan Desert, which is one of the driest places in China, and is characterized by little rainfall and high evapotranspiration rates in excess of ~3000 mm/yr [105–107]. The hyper-arid climate and unique geology of the region result in high soil salinity, and these conditions are excellent for the preservation of organic material such as the Tarim mummies and their associated artifacts [1,30]. Past isotopic research has found correlations between elevated δ^15^N results and environmental factors such as aridity and salinity of the soil [85,86]. Thus, the ^15^N-enriched results of Ganguai, Huoshaogou and the Xinjiang sites are likely influenced by the arid environmental conditions of the Taklamakan Desert (Fig. 4b), in addition to the specific dietary habits of the populations at each site.

The Xinjiang data display robust isotopic evidence for Bronze Age human migration between the cultures of the Eurasian Steppes and the Gansu Corridor and Yellow River Valleys, and confirm this region acted as a bridge between the East and West. This is prominently illustrated at TB, where individual M599 (1940–1765 cal bc) is isotopically indistinguishable from some Gansu Corridor and the Yellow River Valley individuals (Fig. 5), and some of the TB humans plot directly with the south Kazakhstan individuals of Oli-Dzhailau. At TB, the wide range of δ^13^C values indicates that mixed C_3_/C_4_ diets from both the East and the West were consumed (Figs 4a and 5). This site is located ~400 km from Huoshaogou, and is the first major oasis encountered after crossing of the eastern edge of the Taklamakan Desert, which served as a formidable natural geographic barrier for exchanges between the East and West. However, based on the archaeological, anthropological, genetic and dietary evidence (Archaeological Background), a significant population of Di-qiang individuals were living and forming relationships with individuals from the Eurasian Steppe cultures in terms of family, trade and diet since at least 1900 bc, and possibly much earlier [24,69,71].

Yanghai has a late period of occupation compared to the other sites presented (~1200–800 bc), but was included in this survey as the early age range overlaps with the end of the second millennium bc. Like Ganguai, Huoshaogou and TB, the human δ^13^C results at Yanghai also indicate that the diet was a mix of C_3_/C_4_ resources (Fig. 4a) [65]. Yanghai has a wide range of δ^13^C and δ^15^N results that plot with the TB, Karasuk and north Kazakhstan sites (Fig. 5). Thus, the sites of Ganguai, Huoshaogou, TB and Yanghai were likely important incubators for cultural interactions and acted as gateways during the Bronze Age for the movement of people, goods and technologies, both into Central Asia via the Inner Asian Mountain Corridor and into the Yellow River Valleys via the Gansu Corridor.

**Figure 7:**
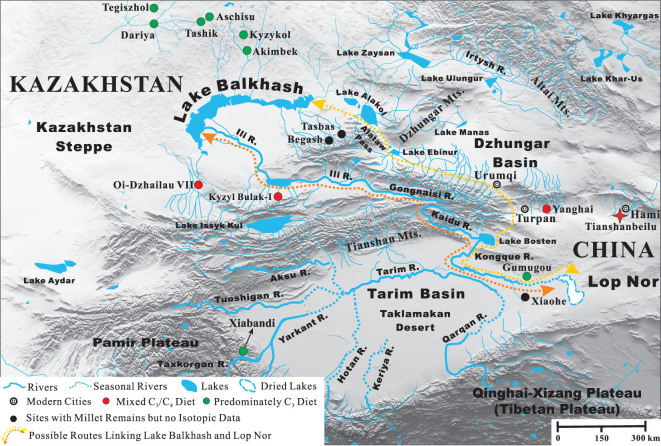
Map showing the possible routes that connected the Lop Nor region and Lake Balkhash. Note the carbon and nitrogen isotopic similarities of Gumugou and north Kazakhstan individuals in Figs 5 and S1 (available as Supplementary Data at *NSR* online).

One of the oldest Bronze Age sites in Xinjiang with substantial archaeological and anthropological evidence related to the Andronovo culture of the Eurasian Steppe is Gumugou (a.k.a. Qäwrighul) [63,64,108,109], and the isotopic results strongly support these links. Located in the Lop Nor region of the eastern part of the Tarim Basin (Fig. 3), the δ^13^C and δ^15^N results of Gumugou are identical to north Kazakhstan sites such as Akimbek, Kyzylkol and Tashik (Figs 5 and S1 (available as Supplementary Data at *NSR* online)). A distance of ~1500 km separates Gumugou from these north Kazakhstan sites, but there is a nearly direct water route of contact from the shores of Lake Balkhash by following the fertile Ili River Valley to the source of the Gongnaisi River in the Tianshan Mountains. From there, it is possible to connect with the Kaidu River and traverse down the southern side of the Tianshan Mountains to Lake Bosten, and then follow the Kongque River to the ancient Lop Nor Lake (dry since the 1960s) (Fig. 7). In support of this possible route, many inhabitants of Gumugou and the closely related site of Xiaohe were found buried with oars, fishing nets and in boat-shaped coffins, indicating that they were accustomed to life around bodies of water [29,31]. However, Andronovo individuals from north Kazakhstan sites may have also traveled to Gumugou and Xiaohe from the north-eastern shores of Lake Balkhash (Fig. 7). This route would have taken them through the Alataw pass and along the northern ridge of the Tianshan Mountains to the modern capital of Xinjiang (Urumqi) and into the Turpan Basin. From here, individuals could have either migrated east to TB or west to Lake Bosten and Lop Nor Lake, and eventually to Gumugou and Xiaohe, and more research (Sr and δ^34^S measurements) is planned to better understand these possible routes of human movement. Millet was not found at Gumugou, and this is supported by the predominately C_3_ human isotopic signatures of the site (Fig. 4a) [63,64]. However, wheat was recovered and a single grain was recently radiocarbon dated to 1890–1750 cal bc [109], which is similar to the results from Huoshaogou [104]. As well as small-scale farming of wheat, the population depended on animal husbandry, hunting and possibly fishing, which, in addition to the arid climate, would also account for the high δ^15^N values and this agrees with the isotopic results (Fig. 4b) [63,64,108].

Xiabandi is located at the far western end of the Tarim Basin and is situated along both sides of the Taxkorgan River (Fig. 3). Based on archaeological and radiocarbon evidence, the site dates to ~1600 bc [110]. The site has strong affiliations with the Andronovo culture, and silver and bronze earrings, identical in design to Eurasian Steppe burials, were found in some graves as well as the remains of goats. No archaeobotanical remains were recovered, but the isotopic results indicate that the population consumed a predominately C_3_ diet [111]. However, six individuals consumed some millet and were possible migrants to the community [111]. The majority of Xiabandi human isotope values overlap with the data of individuals found at sites from north Kazakhstan and the Minusinsk Basin, and the most extreme outlier is similar to the Karasuk population (Fig. 5). Additional research is needed combining isotopic results, direct human radiocarbon dates and ancient DNA to understand the chronology of these dietary patterns in this region, especially at sites along the southern and western portions of the Tarim Basin.

### South Siberia, Russia

The Minusinsk Basin of Russia is located ~1300 km north-west of TB and has a long period of human occupation during the Bronze Age, with sites from the Okunevo (2500–1900 bc), Andronovo (1900–1500 bc) and Karasuk (1500–900 bc) periods. The mean human δ^15^N values from the Minusinsk Basin sites are ~11‰, and the climate is continental and warm/humid with a modern mean precipitation of 341 mm/yr (Fig. 4b). Svyatko *et al.* [9,112] used radiocarbon dates directly paired with δ^13^C values to determine the chronology of when millet first appeared in the human diets of this area. The Okunevo/Andronovo individuals showed no evidence that C_4_ plants were consumed in significant proportions (Fig. 4a). In addition, the Okunevo/Andronovo data of the Minusinsk Basin clusters with the north Kazakhstan sites, especially the western Novoilinovka, Lisakovsk and Bestamak (Figs 3–5 and S1 (available as Supplementary Data at *NSR* online)). This is not surprising given the temporal and cultural similarities between these regions. However, after ~1500 bc, with the onset of the Karasuk culture, there is a rapid change in the δ^13^C values—evidence for a sharp shift to a diet that was a mix of C_3_/C_4_ crops, wheat and millets.

Svyatko *et al.* [9,112] proposed that these millets spread to the Minusinsk Basin from contacts with northern China and, interestingly, the δ^13^C and δ^15^N results of the Karasuk individuals are nearly identical to those from Ganguai, and also isotopically similar to TB, Yanghai, Huoshaogou and Mogou individuals (Fig. 5). The TB results support the work of Svyatko *et al.* [9], since they are isotopically similar in δ^13^C and directly overlap with the same period when the mixed C_3_/C_4_ diets first start to appear at the Karasuk sites. Given the large amount of archaeological material from the Altai Mountains, as well as the anthropological and genetic evidence of admixture between individuals of Siberian and Di-qiang origin [69], it is likely the TB and the Karasuk sites were interacting with each other on a significant scale, despite the distance and barriers of geography (Fig. 1c–e, Archaeological Background). Thus, these results suggest the existence of a Bronze Age ‘Siberian Express’ or a long-distance network of migration and resource transfer that stretched over 2000 km from the Minusinsk Basin of Russia to at least the eastern end of the Gansu Corridor at Mogou 2000 years before the ‘Silk Road’ (Fig. 6 and S2 (available as Supplementary Data at *NSR* online)). That these previously established links rapidly strengthen in the Xinjiang region during the middle portion of the second millennium bc is likely linked to technological advances in horse riding, chariots and metal working, which increased the mobility and migration capabilities of the cultures of the Eurasian Steppe [16,23,113,114]. In China, this period (~1600 bc) is also highly significant, as it marks the establishment of the Shang Dynasty [115]. The observation that these events chronologically coincide has not escaped the notice of past researchers, and there is speculation that increased interaction and exchange with the cultures of the Steppe (e.g. Sintashta-Petrovka, Andronovo, Karasuk, etc.) helped to stimulate the rapid rise and technological advancements of the Shang Dynasty [1,7,23,36,116].

### North Kazakhstan

In contrast, all individuals from Bronze Age sites from the plains of north Kazakhstan (Kyzylkol, Akimbek, Aschisu, Tashik, Dariya, Tegiszhol, Bestamak, Lisakovsk, Novoilinovka) have low δ^13^C values (–19.0‰ to –18.0‰), indicating little or no significant human millet consumption, with diets predominately based on C_3_ crops (Figs 3 and 4a) [13,117,118]. Thus, it appears that these sites were not significantly interacting with the populations of the Yellow River Valleys of China in terms of millet agriculture. However, exceptions exist and some individuals (e.g. Bestamak, B3540; Lisakovsk, L3137, L3155) had δ^13^C values > –18‰ and δ^15^N values > 13‰ and an outlier from Tegiszhol overlaps with three individuals from TB (Fig. 5). Ventresca Miller *et al.* [117] suggested that the Bestamak and Lisakovsk individuals might have consumed more fish or C_4_ plants in relation to the other members of these sites, so there is the possibility of some migrants from Xinjiang in these populations (note the isotopic similarities with Gumugou, Fig. 5 and S1 (available as Supplementary Data at *NSR* online)) and future genetic research is needed. In north Kazakhstan, the δ^15^N values are elevated between ~11‰ to 14‰ but, compared to south Siberia (~340–420 mm/yr), there is a general decrease in modern mean precipitation (~310–320 mm/yr), and the climate is warm, humid and continental. As with Xinjiang, these high human δ^15^N values were likely influenced by the environmental factors as well as the specific diets of these populations (Fig. 4b), and future isotopic analysis of archaeological plant remains will provide more information on the baseline isotopic values in this region.

### South Kazakhstan and Central Asia

The south Kazakhstan sites of Kyzyl Bulak and Oi-Dzhailau display a large range of isotopic results and are located along the northern slope of the Tianshan Mountains (Fig. 3), approximately 2000 km from TB [13]. Like TB, the inhabitants at both of these sites had similar δ^13^C values, indicative of mixed C_3_/C_4_ diets and significant millet consumption (Fig. 4a) [13]. The human δ^15^N values at Kyzyl Bulak (10.7‰) and Oi-Dzhailau (13.8‰) show large differences even though they have similar climates (hot humid continental). While the modern mean rainfall is somewhat different between these sites (Fig. 4b), the isotopic difference (3.1‰) represents approximately one trophic level and was more likely influenced by dietary preferences than environmental conditions. Three Kyzyl Bulak individuals are similar to the isotopic values from the Minusinsk Basin, and the fourth plots near TB and Oi-Dzhailau (Fig. 5). In contrast, the Oi-Dzhailau data overlap with TB, but two individuals cluster with the north Kazakhstan populations, and this is possible evidence that migration and exchange occurred between the sites of north and south Kazakhstan as well as TB, but genetic analysis is necessary to confirm this. In addition, Kyzyl Bulak and Oi-Dzhailau date to the same general time period (1750–1400 cal bc) as TB (Fig. S2, available as Supplementary Data at *NSR* online). This is strong evidence in support of the Inner Asian Mountain Corridor as an early main conduit for the spread of millet (as well as cultural and technological exchanges and migration) from the Yellow River Valleys via Xinjiang to south Kazakhstan (Figs 6 and S2 (available as Supplementary Data at *NSR* online)) [6].

Continuing west along the Inner Asian Mountain Corridor, directly dated millet grains have also been found during the same timeframe at Ojakly (1740–1610 cal bc) in eastern Turkmenistan [6,11,35,119] (Fig. 6). This finding, as well as the discovery of small bundles of ephedra (*Ephedra* sp.) with the Tarim mummies at sites such as Gumugou and Xiaohe, suggests interactions with the Bactria–Margiana Archaeological Complex (2300–1700 bc) of the Iranian Plateau [1,23,30]. In addition, archaeological finds of millet have been reported from Shortughai in northern Afghanistan [6,120,121] and at many other sites in Iran, Iraq, Syria and Turkey during the second millennium bc, but these grains were not directly radiocarbon dated [8]. Thus, we hypothesize that future isotopic studies of Bronze Age human populations in Kyrgyzstan, Tajikistan, Uzbekistan, Turkmenistan, Afghanistan, Pakistan, India, Iran, Iraq and Turkey will uncover evidence of mixed C_3_/C_4_ human diets, and thus directly extend the Isotopic Millet Road from its current terminus in southern Kazakhstan (Fig. 6).

### Europe

In Bronze Age Europe, human bone collagen δ^13^C values that could be interpreted as evidence of millet consumption are found at sites in Ukraine (Glubokoe Ozero II), northern Greece (Rymnio) and in northern (Olmo di Nogara) and central Italy (Grotta Misa, Felcetone) [122–126] (Fig. 6). Of these locations, the Italian site of Olmo di Nogara [107] currently displays the most convincing evidence that a European population consumed millet in similar amounts to the individuals at Oi-Dzhailau and TB (Figs 4a and 5). The Olmo di Nogara individuals are believed to date (no radiocarbon dates were performed) to the same general period (~1600–1200 bc) as the sites in south Kazakhstan, Siberia and China, and it is possible that these sites might have had links in terms of human connections such as migration and resource transfer. This could have been facilitated by a communication network maintained between the Srubnaya and Andronovo cultures of the western and eastern Eurasian Steppes, respectively [23], and future research (radiocarbon dating, ancient DNA and archaeobotanical analysis) at Olmo di Nogara is needed.

Millet was also recovered at many other Bronze Age sites in Europe (Fig. 6) [32,42,127–129] but, as few of these grains were directly radiocarbon dated, the inferred dates based on associated materials are less secure [42]. However, the direct radiocarbon dating of millet grains revealed ages (1610–1260 cal bc) at the sites of Fajsz18 (Hungry), Bruchenbrücken/Friedberg (Germany) and Măgura-Buduiasca (Romania) that overlap with the dates from the sites of south Kazakhstan, Minusinsk Basin and Xinjiang (Figs 6 and S2 (available as Supplementary Data at *NSR* online)) [42]. Further, a millet grain was directly dated at Assiros in Greece (1395–1285 cal bc) [130] and Valamoti [127] reported an earlier radiocarbon date of 2200–2030 cal bc for broomcorn millet grains from the Greek site of Skala Sotiros. In addition, millet was recently directly radiocarbon dated at Qasim Bagh (1495-1305 cal BC) in India [131] and Guamsky Grot (1110-910 cal BC) in Russia [132]. While these results are intriguing, additional isotopic, genetic and archaeological studies of Bronze Age sites (from eastern/southern Europe, Central Asia and Russia) are required to confirm that this Isotopic Millet Road stretched directly from the Yellow River Valleys of China to Europe and to understand what this form of communication/interaction may have meant to these distant populations.

## CONCLUSIONS

As a period characterized by dynamic East–West migrations, the second millennium bc of Eurasia witnessed frequent exchanges of crops, animals, tools, as well as ideas among different populations. Accompanying these interactions, extensive changes in daily life and social structures occurred in the early civilizations across the Eurasian continent, including both ancient China and the Mediterranean. In particular, China experienced a rapid increase in human population and settlements from the late Neolithic to the Bronze Age [7,115], and this set the stage for the ‘revolutionary’ development of the Chinese civilization and the formation of early states in China. It is in this context and during this critical transitional period that millet agriculture/consumption started to significantly expand westward out of the Yellow River Valleys and appear in Xinjiang, south Kazakhstan, Minusinsk Basin and Europe by the Isotopic Millet Road. We suggest that increased East–West contact, in the form of resource transfer, warfare, marriage, migration and the rise of the Xia and Shang Dynasties, was a major driver for this Bronze Age radiation of millets. Thus, the isotopic results presented here, combined with the archaeology, physical anthropology and genetic evidence, indicate that sites in Xinjiang (such as TB) held unique positions between the Bronze Age cultures of the East and West with the direct mixing of many aspects of daily life, from family to foods.

## Supplementary Material

nwx015_Supplement_File
