# Supplementary material for: Tianshanbeilu and the Isotopic Millet Road: reviewing the late Neolithic/Bronze Age radiation of human millet consumption from north China to Europe
Source: Natl Sci Rev. 2017 Feb 24;6(5):1024–39. doi: 10.1093/nsr/nwx015 (PMC8291513; doi:10.1093/nsr/nwx015)
Supplement: nwx015_Supplement_File [file nwx015_supplement_file.docx]

**Supporting Information**

**
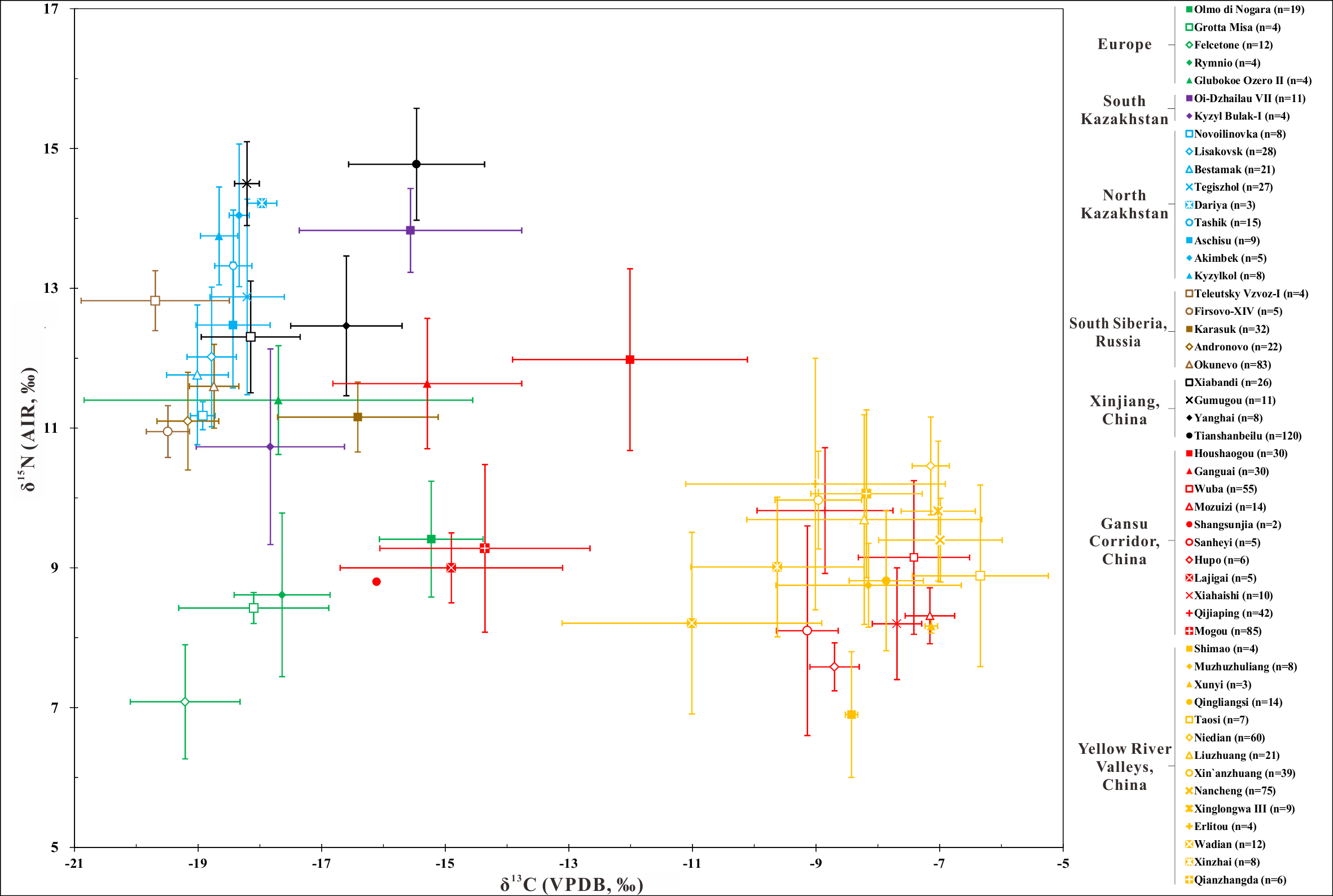
**

**Figure S1.** Mean ± SD plot showing the isotopic results of Bronze Age populations across Eurasia.

**
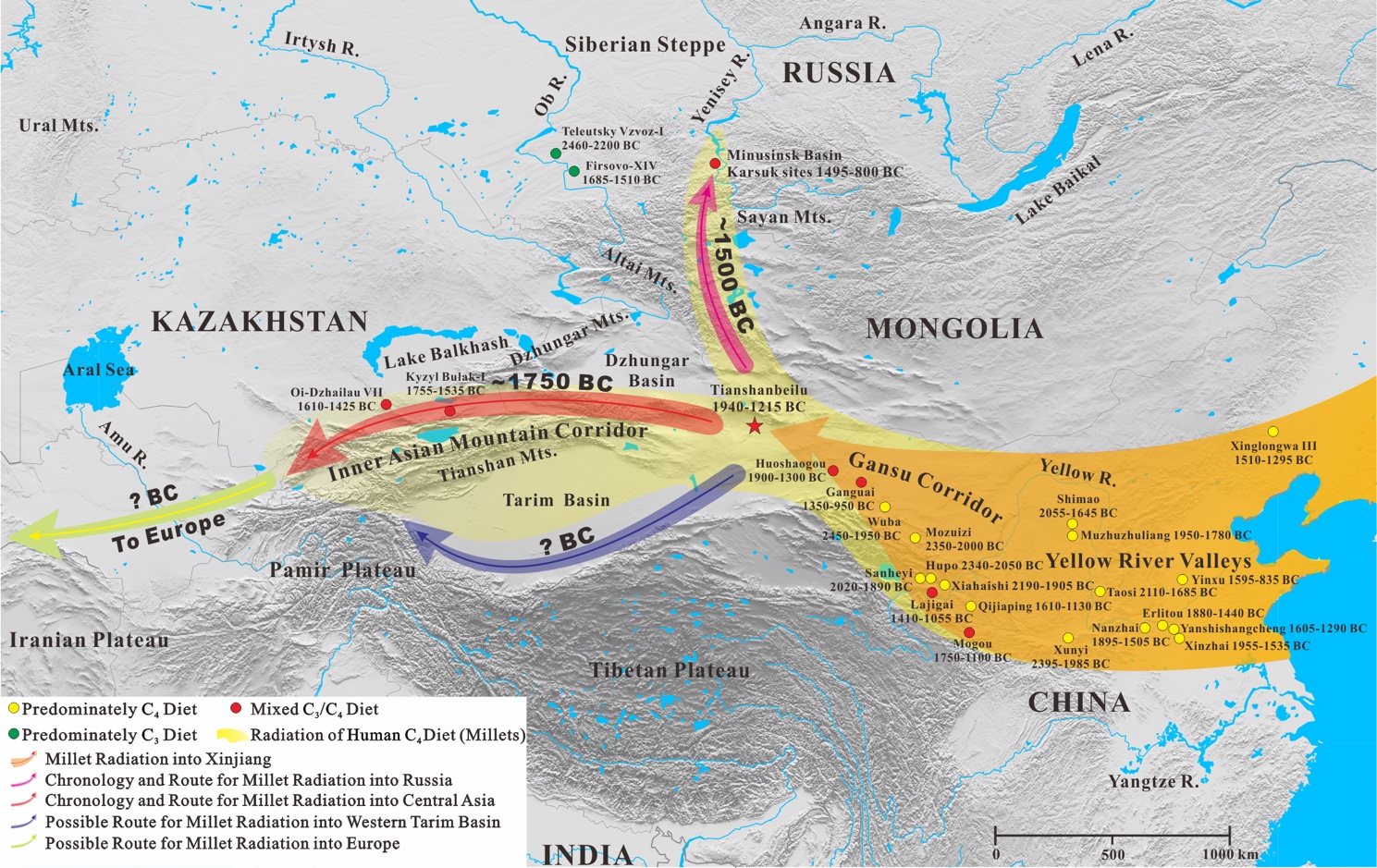
**

**Figure S2.** Map showing sites from the Yellow River Valleys, Gansu Corridor, Xinjiang, Kazakhstan and southern Siberia where human remains were directly radiocarbon dated to visualize the chronology of the westward radiation of human millet consumption. Results and references presented in Table S2.

Table S1. Sample information and isotope data of the human and animal bones from the Tianshanbeilu cemetery, Xinjiang, China.

| **Sample No.** | **Burial Context** | **Age** | **Sex** | **Bone Type** | **Isotopic Data** | | | | | | | **Carbon Dating** | |
| --- | --- | --- | --- | --- | --- | --- | --- | --- | --- | --- | --- | --- | --- |
|  |  |  |  |  | **Yield** | **δ^13^C**  **(‰)** | **δ^15^N (‰)** | **(%)C** | **(%)N** | **Atomic C:N** | **LAB*** | **Beta Code** | **Uncalibrated ^14^C Ages（BP）** |
| T33 | M425 | sheep/goat | ? | Femur | 9.4 | -17.8 | 8.7 | 41.3 | 15.4 | 3.1 | ESIL | **-** | **-** |
|  |  |  |  |  |  |  |  |  |  |  |  |  |  |
| T11 | T3M3 | Adult | ? | Femur | 10.6 | -16.7 | 13.0 | 41.8 | 15.6 | 3.1 | ESIL | - | - |
| T13 | T3M12B | Adult | ? | Femur | 16.0 | -14.5 | 13.5 | 44.0 | 16.1 | 3.2 | ESIL | - | - |
| TB1 | T3M15:B | Adult | ? | Cranium | 6.4 | -13.5 | 14.8 | 43.6 | 16.2 | 3.2 | ASIL | - | - |
| T14 | T10M2 | Adult | ? | Femur | 15.6 | -15.7 | 15.0 | 41.8 | 15.7 | 3.1 | ESIL | - | - |
| TB2 | T10M2:1 | Adult | ? | Cranium | 6.0 | -15.7 | 14.9 | 43.8 | 16.4 | 3.1 | ASIL | - | - |
| TB3 | T12M5 | Adult | ? | Cranium | 7.4 | -15.0 | 13.9 | 36.7 | 13.9 | 3.1 | ASIL | - | - |
| TB4 | T12M11 | Adult | ? | Cranium | 3.5 | -15.1 | 15.3 | 44.8 | 16.6 | 3.1 | ASIL | - | - |
| TB5 | T14M5 | Adult | ? | Cranium | 3.9 | -15.8 | 15.1 | 39.8 | 14.9 | 3.1 | ASIL | - | - |
| T3 | T14M7 | Adult | ? | Femur | 12.8 | -15.7 | 15.0 | 43.9 | 16.1 | 3.2 | ESIL | - | - |
| T8 | T14M8 | Adult | ? | Femur | 7.8 | -16.9 | 14.8 | 41.9 | 15.4 | 3.2 | ESIL | - | - |
| T7 | T14M14 | Adult | ? | Femur | 10.5 | -15.1 | 14.4 | 41.1 | 15.3 | 3.1 | ESIL | - | - |
| T9 | T14M16 | Adult | ? | Femur | 7.1 | -15.7 | 14.5 | 41.5 | 15.2 | 3.2 | ESIL | - | - |
| TB6 | T16M2:A | Adult | ? | Cranium | 5.5 | -15.4 | 15.4 | 40.1 | 15.0 | 3.1 | ASIL | - | - |
| TB7 | T16M11 | Adult | ? | Cranium | 2.3 | -15.3 | 14.8 | 40.7 | 15.3 | 3.1 | ASIL | - | - |
| TB8 | T16M12 | Adult | ? | Cranium | 4.1 | -15.7 | 15.0 | 42.3 | 15.8 | 3.1 | ASIL | - | - |
| TB9 | T16M18 | Adult | ? | Cranium | 4.1 | -16.9 | 16.1 | 40.3 | 15.0 | 3.1 | ASIL | - | - |
| TB10 | T18M15 | Adult | ? | Mandible | 1.6 | -15.4 | 14.6 | 39.7 | 14.9 | 3.1 | ASIL | - | - |
| TB11 | T18M17 | Adult | ? | Cranium | 1.7 | -15.5 | 14.9 | 42.0 | 15.8 | 3.1 | ASIL | - | - |
| T6 | T21M4 | Adult | ? | Femur | 7.6 | -16.8 | 15.4 | 43.0 | 15.9 | 3.2 | ESIL | - | - |
| TB12 | T25M8 | Adult | ? | Metacarpus | 2.8 | -15.3 | 14.4 | 37.4 | 14.0 | 3.2 | ASIL | - | - |
| TB13 | T25M45 | Adult | ? | Cranium | 8.0 | -15.6 | 15.6 | 39.7 | 14.8 | 3.1 | ASIL | - | - |
| T4 | T30M1 | Adult | ? | Femur | 6.9 | -16.5 | 14.8 | 41.7 | 15.6 | 3.1 | ESIL | - | - |
| TB14 | T30M2 | Adult | ? | Cranium | 2.3 | -15.5 | 15.1 | 41.2 | 15.5 | 3.1 | ASIL | - | - |
| T10 | T33M1 | Adult | ? | Femur | 9.4 | -16.1 | 14.7 | 42.5 | 15.6 | 3.2 | ESIL | - | - |
| T12 | T33M2 | Adult | ? | Femur | 8.8 | -16.5 | 14.7 | 43.3 | 15.9 | 3.2 | ESIL | - | - |
| T5 | T34M8 | Adult | female | Femur | 10.0 | -15.4 | 14.4 | 41.3 | 15.2 | 3.2 | ESIL | - | - |
| TB15 | T35M1 | Adult | ? | Cranium | 2.0 | -15.2 | 13.6 | 39.5 | 14.9 | 3.1 | ASIL | - | - |
| T2 | T35M2 | Adult | ? | Femur | 4.3 | -15.6 | 13.6 | 42.6 | 15.7 | 3.2 | ESIL | - | - |
| T1 | T35M4 | Adult | ? | Femur | 12.3 | -13.6 | 13.6 | 42.8 | 15.7 | 3.2 | ESIL | Beta-416251 | 3230±30 |
| T15 | M9 | Adult | male | Femur | 15.1 | -16.4 | 14.2 | 41.6 | 15.4 | 3.1 | ESIL | - | - |
| T17 | M13A | Adult | ? | Femur | 14.4 | -16.4 | 14.9 | 43.1 | 15.8 | 3.2 | ESIL | - | - |
| T16 | R14 | Adult | ? | Femur | 8.4 | -15.5 | 14.9 | 41.6 | 15.5 | 3.1 | ESIL | - | - |
| T18 | M17 | Adult | ? | Femur | 19.0 | -16.2 | 14.3 | 43.1 | 16.0 | 3.1 | ESIL | - | - |
| TB16 | M12 | Adult | ? | Mandible | 9.1 | -16.5 | 15.3 | 43.0 | 16.0 | 3.1 | ASIL | - | - |
| TB17 | M84 | Adult | ? | Cranium | 5.2 | -14.6 | 14.9 | 41.9 | 15.6 | 3.1 | ASIL | - | - |
| TB18 | M206(T25M12) | Adult | ? | Mandible | 5.1 | -15.6 | 15.1 | 43.3 | 16.2 | 3.1 | ASIL | - | - |
| TB19 | M219 | Adult | ? | Mandible | 4.9 | -16.2 | 14.2 | 41.1 | 15.5 | 3.1 | ASIL | - | - |
| TB20 | M230B | Adult | ? | Cranium | 7.6 | -17.6 | 15.7 | 43.1 | 16.1 | 3.1 | ASIL | - | - |
| TB21 | M253(T31M4) | Adult | ? | Cranium | 3.1 | -14.3 | 15.2 | 42.8 | 16.0 | 3.1 | ASIL | - | - |
| TB22 | M284(T34M6) | Adult | ? | Mandible | 3.7 | -12.8 | 13.4 | 42.9 | 16.0 | 3.1 | ASIL | - | - |
| TB23 | M311 | Adult | ? | Cranium | 3.5 | -16.1 | 15.4 | 39.3 | 14.9 | 3.1 | ASIL | - | - |
| T21 |  |  |  | Femur | 10.0 | -16.5 | 14.5 | 42.6 | 15.8 | 3.1 | ESIL | - | - |
| T28 | M312A | Adult | male | Femur | 6.3 | -15.9 | 15.1 | 42.8 | 15.8 | 3.2 | ESIL | - | - |
| TB24 | M315 | Adult | ? | Cranium | 1.8 | -16.3 | 15.1 | 42.2 | 15.8 | 3.1 | ASIL | - | - |
| T19 |  |  |  | Femur | 8.0 | -16.9 | 14.4 | 41.5 | 15.3 | 3.2 | ESIL | Beta-416252 | 3190±30 |
| T20 | M316 | Adult | ? | Femur | 11.3 | -16.8 | 14.6 | 41.6 | 15.5 | 3.1 | ESIL | - | - |
| TB25 | M317 | Adult | female | Mandible | 12.0 | -15.9 | 15.5 | 45.1 | 16.7 | 3.1 | ASIL | - | - |
| T27 |  |  |  | Femur | 14.6 | -16.4 | 15.0 | 44.0 | 16.2 | 3.2 | ESIL | - | - |
| TB26 | M321 | Adult | ? | Cranium | 8.8 | -14.8 | 13.1 | 41.3 | 15.5 | 3.1 | ASIL | - | - |
| T25 | M323 | Adult | ? | Femur | 6.0 | -15.5 | 13.9 | 41.4 | 15.6 | 3.1 | ESIL | - | - |
| T22 | M329 | Adult | ? | Femur | 4.7 | -15.4 | 14.3 | 39.6 | 14.9 | 3.1 | ESIL | - | - |
| TB27 | M330 | Adult | ? | Cranium | 4.2 | -14.9 | 15.2 | 44.3 | 16.5 | 3.1 | ASIL | - | - |
| T24 | M336 | Adult | ? | Femur | 8.9 | -16.2 | 14.5 | 40.9 | 15.5 | 3.1 | ESIL | - | - |
| T23 | M337 | Adult | ? | Femur | 8.3 | -15.6 | 15.1 | 40.5 | 15.3 | 3.1 | ESIL | - | - |
| TB28 | M358 | Adult | ? | Cranium | 6.2 | -16.0 | 14.1 | 42.8 | 16.0 | 3.1 | ASIL | - | - |
| T26 | M354 | Adult | male | Femur | 9.8 | -16.2 | 15.1 | 41.4 | 15.5 | 3.1 | ESIL | Beta-419186 | 3040±30 |
| TB29 | M371(owner) | Adult | ? | Cranium | 9.0 | -15.8 | 15.0 | 45.6 | 17.0 | 3.1 | ASIL | - | - |
| TB30 | M377 | Adult | ? | Cranium | 4.2 | -16.1 | 14.6 | 44.1 | 16.5 | 3.1 | ASIL | - | - |
| TB31 | M390 | Adult | ? | Cranium | 5.8 | -16.6 | 14.0 | 43.3 | 16.1 | 3.1 | ASIL | - | - |
| T29 | M391 | Adult | ? | Femur | 14.8 | -14.9 | 14.3 | 43.7 | 16.1 | 3.2 | ESIL | - | - |
| T30 | M394 | Adult | female | Femur | 12.2 | -15.6 | 14.5 | 43.7 | 16.8 | 3.2 | ESIL | - | - |
| TB32 | M425 | Adult | ? | Cranium | 7.0 | -15.5 | 16.1 | 43.7 | 16.4 | 3.1 | ASIL | - | - |
| TB33 | M429 | Adult | ? | Cranium | 4.6 | -14.7 | 15.9 | 44.8 | 16.7 | 3.1 | ASIL | - | - |
| TB34 | M436A | Adult | ? | Cranium | 14.2 | -15.8 | 15.7 | 44.5 | 16.5 | 3.1 | ASIL | - | - |
| TB35 | M446:A | Adult | ? | Cranium | 6.9 | -16.5 | 13.0 | 44.3 | 16.6 | 3.1 | ASIL | - | - |
| TB36 | M448:A | Adult | ? | Cranium | 13.8 | -15.8 | 15.1 | 44.9 | 16.5 | 3.2 | ASIL | - | - |
| TB37 | M455(owner) | Adult | ? | Pelvis | 14.6 | -15.7 | 14.7 | 42.2 | 15.8 | 3.1 | ASIL | - | - |
| TB38 | M450:A | Adult | ? | Cranium | 7.4 | -15.6 | 14.7 | 43.4 | 16.2 | 3.1 | ASIL | - | - |
| TB39 | M459 | Adult | ? | Cranium | 13.6 | -16.3 | 15.0 | 44.3 | 16.5 | 3.1 | ASIL | - | - |
| TB40 | M460 | Adult | ? | Cranium | 11.0 | -14.2 | 14.8 | 42.0 | 15.6 | 3.1 | ASIL | - | - |
| TB41 | M471:A | Adult | ? | Cranium | 7.7 | -14.1 | 14.3 | 44.1 | 16.6 | 3.1 | ASIL | - | - |
| TB42 | M472 | Adult | ? | Cranium | 10.5 | -15.1 | 15.9 | 44.6 | 16.7 | 3.1 | ASIL | - | - |
| TB43 | M473 | Adult | ? | Cranium | 16.2 | -14.8 | 15.5 | 44.5 | 16.6 | 3.1 | ASIL | - | - |
| TB44 | M474 | Adult | ? | Cranium | 8.1 | -13.5 | 14.6 | 39.7 | 14.9 | 3.1 | ASIL | - | - |
| TB45 | M475 | Adult | ? | Cranium | 6.2 | -15.1 | 15.2 | 43.6 | 16.1 | 3.1 | ASIL | - | - |
| TB46 | M479 | Adult | ? | Cranium | 11.4 | -14.4 | 13.6 | 47.4 | 17.8 | 3.1 | ASIL | - | - |
| TB47 | M480 | Adult | ? | Mandible | 9.1 | -15.1 | 14.5 | 44.0 | 16.5 | 3.1 | ASIL | - | - |
| TB48 | M483 | Adult | ? | Mandible | 4.0 | -16.5 | 15.5 | 36.0 | 13.5 | 3.1 | ASIL | - | - |
| TB49 | M487:A | Adult | ? | Cranium | 6.7 | -16.1 | 14.6 | 40.3 | 15.2 | 3.1 | ASIL | - | - |
| TB50 | M488 | Adult | ? | Cranium | 13.3 | -15.9 | 14.5 | 44.3 | 16.6 | 3.1 | ASIL | - | - |
| TB51 | M492 | Adult | ? | Cranium | 11.1 | -15.8 | 16.7 | 45.8 | 17.1 | 3.1 | ASIL | - | - |
| TB52 | M500(owner) | Adult | ? | Cranium | 2.0 | -16.3 | 15.0 | 38.2 | 14.5 | 3.1 | ASIL | - | - |
| TB53 | M503 | Adult | ? | Humerus | 15.5 | -14.6 | 14.4 | 43.5 | 16.2 | 3.1 | ASIL | - | - |
| TB54 | M503:A | Adult | ? | Mandible | 10.2 | -14.6 | 14.6 | 40.5 | 14.9 | 3.2 | ASIL | - | - |
| TB55 | M505 | Adult | ? | Mandible | 11.7 | -13.9 | 14.9 | 43.1 | 16.1 | 3.1 | ASIL | - | - |
| TB56 | M511 | Adult | ? | Mandible | 4.4 | -14.8 | 14.1 | 36.4 | 13.5 | 3.1 | ASIL | - | - |
| TB85 | M518 | Adult | ? | Cranium | 1.8 | -15.9 | 15.7 | 38.3 | 14.4 | 3.1 | ASIL | - | - |
| TB57 | M528 | Adult | ? | Mandible | 8.1 | -14.2 | 14.2 | 39.7 | 14.9 | 3.1 | ASIL | - | - |
| TB58 | M529:A | Adult | ? | Mandible | 14.1 | -15.6 | 15.1 | 41.5 | 15.4 | 3.1 | ASIL | - | - |
| TB86 | M530 | Adult | ? | Cranium | 13.4 | -15.2 | 14.4 | 41.9 | 15.5 | 3.1 | ASIL | - | - |
| TB59 | M532 | Adult | ? | Mandible | 13.8 | -15.2 | 15.2 | 43.6 | 16.3 | 3.1 | ASIL | - | - |
| TB60 | M542 | Adult | ? | Cranium | 11.0 | -14.2 | 14.5 | 42.1 | 15.8 | 3.1 | ASIL | - | - |
| TB61 | M554 | Adult | ? | Cranium | 13.1 | -14.9 | 15.0 | 43.7 | 16.2 | 3.1 | ASIL | - | - |
| TB62 | M554:A | Adult | ? | Mandible | 11.8 | -16.5 | 15.7 | 42.5 | 15.8 | 3.1 | ASIL | - | - |
| TB63 | M556:A | Adult | ? | Cranium | 12.3 | -15.4 | 15.8 | 45.0 | 16.8 | 3.1 | ASIL | - | - |
| TB66 | M571 | Adult | ? | Cranium | 15.2 | -15.3 | 16.6 | 42.3 | 15.9 | 3.1 | ASIL | - | - |
| TB67 | M588 | Adult | ? | Maxillary | 6.8 | -15.3 | 14.7 | 38.7 | 14.4 | 3.1 | ASIL | - | - |
| TB68 | M590 | Adult | ? | Cranium | 13.0 | -16.3 | 14.8 | 42.5 | 15.9 | 3.1 | ASIL | - | - |
| TB69 | M599 | Adult | ? | Cranium | 13.2 | -7.0 | 10.5 | 43.0 | 16.1 | 3.1 | ASIL | Beta-429482 | 3530±30 |
| TB70 |  |  |  | Humerus | 3.8 | -7.4 | 10.1 | 36.9 | 13.7 | 3.1 | ASIL | - | - |
| TB71 | M602 | Adult | ? | Cranium | 13.8 | -16.3 | 14.7 | 43.7 | 16.3 | 3.1 | ASIL | - | - |
| TB72 | M611(owner) | Adult | ? | Mandible | 13.3 | -15.4 | 13.6 | 43.4 | 16.2 | 3.1 | ASIL | - | - |
| TB73 | M612 | Adult | ? | Cranium | 12.4 | -15.2 | 14.9 | 44.7 | 16.8 | 3.1 | ASIL | - | - |
| TB74 | M622 | Adult | ? | Maxillary | 10.9 | -15.5 | 14.8 | 39.9 | 14.9 | 3.1 | ASIL | - | - |
| TB75 | M622:A | Adult | ? | Cranium | 11.9 | -15.8 | 14.8 | 46.0 | 17.1 | 3.1 | ASIL | - | - |
| TB76 | M636 | Adult | ? | Cranium | 12.0 | -15.4 | 14.4 | 41.5 | 15.5 | 3.1 | ASIL | - | - |
| TB77 | M652 | Adult | ? | Cranium | 7.6 | -16.4 | 14.8 | 36.4 | 13.7 | 3.1 | ASIL | - | - |
| TB78 | M653 | Adult | ? | Cranium | 11.0 | -16.0 | 15.1 | 42.3 | 15.8 | 3.1 | ASIL | - | - |
| TB79 | M660 | Adult | ? | Cranium | 9.6 | -14.6 | 14.0 | 39.0 | 14.6 | 3.1 | ASIL | - | - |
| TB80 | M673 | Adult | ? | Mandible | 11.5 | -16.5 | 15.3 | 43.3 | 16.2 | 3.1 | ASIL | - | - |
| TB81 | M676 | Adult | ? | Mandible | 10.6 | -15.7 | 15.4 | 42.1 | 15.7 | 3.1 | ASIL | - | - |
| TB82 | M683 | Adult | ? | Mandible | 9.8 | -17.4 | 12.1 | 39.8 | 14.8 | 3.1 | ASIL | - | - |
| TB83 | M696 | Adult | ? | Cranium | 13.0 | -15.7 | 15.8 | 40.0 | 15.1 | 3.1 | ASIL | - | - |
| TB84 | M703 | Adult | ? | Cranium | 12.2 | -15.2 | 15.4 | 38.3 | 14.5 | 3.1 | ASIL | - | - |

* ESIL= The Environment Stable Isotope Lab of the Chinese Academy of Agricultural Sciences; ASIL = The Archaeological Stable Isotope Lab of the University of Chinese Academy of Sciences.

Table S2. Review of published δ^13^C and δ^15^N values for humans (≥ 3) from Bronze Age sites in north China and Central Asia as well as sites with millet consumption from Europe.

| **Location** | **Site name** | **Age**  **(BC)** | **^14^C dates** | | | **Isotopic Analysis** | | | | | | **References** |
| --- | --- | --- | --- | --- | --- | --- | --- | --- | --- | --- | --- | --- |
|  |  |  | **No.** | **Sample Type** | **Calibrated Age (Cal. BC; 2σ)** | **No.** | **Sample Type** | **δ^13^C (‰)** | **SD** | **δ^15^N (‰)** | **SD** |  |
| **Europe** | Olmo di Nogara | 1600-1100 |  |  |  | 19 | bone collagen | -15.2 | 0.8 | 9.4 | 0.8 | [[124](#_ENREF_124)] |
|  | Grotta Misa | 1700-1350 |  |  |  | 4 | bone collagen | -18.1 | 1.2 | 8.4 | 0.2 | [[126](#_ENREF_126)] |
|  | Felcetone | 1700-1350 |  |  |  | 12 | bone collagen | -19.2 | 0.9 | 7.1 | 0.8 | [[126](#_ENREF_126)] |
|  | Rymnio | 1700-1100 |  |  |  | 4 | bone collagen | -17.6 | 0.8 | 8.6 | 1.2 | [[122](#_ENREF_122)] |
|  | Glubokoe Ozero Ⅱ | 1400-1200 |  |  |  | 4 | bone collagen | -17.7 | 3.1 | 11.4 | 0.8 | [[123](#_ENREF_123)] |
| **South Kazakhstan** | Oi-Dzhailau Ⅶ | 1610-1425 | 2 | bone collagen | 1610-1425 | 11 | bone collagen | -15.6 | 1.8 | 13.8 | 0.6 | [[13](#_ENREF_13)] |
|  | Kyzyl Bulak-Ι | 1755-1535 | 2 | bone collagen | 1755-1535 | 4 | bone collagen | -17.8 | 1.2 | 10.7 | 1.4 | [[13](#_ENREF_13)] |
| **North Kazakhstan** | Novoilinovka | 1900-1600 |  |  |  | 8 | bone collagen | -18.9 | 0.2 | 11.2 | 0.2 | [[13](#_ENREF_13)] |
|  | Lisakovsk | 1860-1680 |  |  |  | 28 | bone collagen | -18.8 | 0.4 | 12.0 | 1.0 | [[117](#_ENREF_117)] |
|  | Bestamak | 2030-1640 |  |  |  | 21 | bone collagen | -19.0 | 0.5 | 11.8 | 1.0 | [[117](#_ENREF_117)] |
|  | Tegiszhol | 1800-700 |  |  |  | 27 | bone collagen | -18.2 | 0.6 | 12.9 | 1.4 | [[118](#_ENREF_118)] |
|  | Dariya | 1700-1200 |  |  |  | 3 | bone collagen | -18.0 | 0.2 | 14.2 | 0 | [[118](#_ENREF_118)] |
|  | Tashik | 1700-1500 |  |  |  | 15 | bone collagen | -18.4 | 0.3 | 13.3 | 0.8 | [[118](#_ENREF_118)] |
|  | Aschisu | 1800-1600 |  |  |  | 9 | bone collagen | -18.4 | 0.6 | 12.5 | 0.9 | [[118](#_ENREF_118)] |
|  | Akimbek | 1700-1200 |  |  |  | 5 | bone collagen | -18.3 | 0.2 | 14.0 | 1.0 | [[118](#_ENREF_118)] |
|  | Kyzylkol | 1800-1700 |  |  |  | 8 | bone collagen | -18.7 | 0.3 | 13.7 | 0.7 | [[118](#_ENREF_118)] |
| **South Siberia, Russia** | Teleutsky Vzvoz-I | 2460-2200 | 1 | bone collagen | 2460-2200 | 4 | bone collagen | -19.7 | 1.2 | 12.8 | 0.4 | [[116](#_ENREF_116)] |
|  | Firsovo-XIV | 1685-1510 | 1 | bone collagen | 1685-1510 | 5 | bone collagen | -19.5 | 0.3 | 11.0 | 0.4 | [[116](#_ENREF_116)] |
|  | Karasuk sites | 1500-900 | 25 | bone collagen | 1495-800 | 32 | bone collagen | -16.4 | 1.3 | 11.4 | 0.5 | [[9](#_ENREF_9), [112](#_ENREF_112)] |
|  | Andronovo sites | 1900-1500 | 14 | bone collagen | 1765-1400 | 22 | bone collagen | -19.2 | 0.5 | 11.2 | 0.6 | [[9](#_ENREF_9), [112](#_ENREF_112)] |
|  | Okunevo sites | 2500-1900 | 21 | bone collagen | 2620-1745 | 83 | bone collagen | -18.7 | 0.4 | 11.6 | 0.6 | [[9](#_ENREF_9), [112](#_ENREF_112)] |
| **Xinjiang, China** | Xiabandi | 1950-1300 |  |  |  | 26 | bone collagen | -18.1 | 0.8 | 12.3 | 1.0 | [[111](#_ENREF_111)] |
|  | Gumugou | 1900-1500 |  |  |  | 11 | bone collagen | -18.2 | 0.2 | 14.5 | 0.6 | [[63](#_ENREF_63), [64](#_ENREF_64)] |
|  | Yanghai | 1200-800 |  |  |  | 8 | bone collagen | -16.6 | 0.9 | 12.5 | 1.0 | [[65](#_ENREF_65)] |
|  | Tianshanbeilu | 2000-1300 | 4 | bone collagen | 1940-1215 | 120 | bone collagen | -15.5 | 1.1 | 14.8 | 0.8 | This study; [[66](#_ENREF_66)] |
| **Gansu Corridor, China** | Huoshaogou | 1900-1300 | 8 | bone collagen | 1900-1300 | 30 | bone collagen | -12.0 | 1.9 | 12.0 | 1.3 | [[44](#_ENREF_44)] |
|  | Ganguai | 1350-950 | 1 | bone collage | 1350-950 | 30 | bone collagen | -15.3 | 1.5 | 11.6 | 0.9 | [[44](#_ENREF_44)] |
|  | Wuba | 2450-1950 | 4 | bone collagen | 2450-1950 | 55 | bone collagen | -7.4 | 0.9 | 9.1 | 1.1 | [[44](#_ENREF_44)] |
|  | Mozuizi | 2350-2000 | 4 | bone collagen | 2350-2000 | 14 | bone collagen | -7.2 | 0.4 | 8.3 | 0.4 | [[44](#_ENREF_44)] |
|  | Shangsunjia | 1300-1000 |  |  |  | 18/2 | bone collagen | -16.1 | 1.3 | 8.8 | 0.8 | [[88](#_ENREF_88)] |
|  | Sanheyi | ca. 2000 | 1 | bone collagen | 2020-1890* | 5 | bone collagen | -9.1 | 0.5 | 8.1 | 1.5 | [[101](#_ENREF_101)] |
|  | Hupo | ca. 2000 | 1 | bone collagen | 2340-2050* | 6 | bone collagen | -8.7 | 0.4 | 7.5 | 0.3 | [[101](#_ENREF_101)] |
|  | Lajigai | ca. 1300 | 1 | bone collagen | 1410-1055* | 5 | bone collagen | -14.9 | 1.8 | 9 | 0.5 | [[101](#_ENREF_101)] |
|  | Xiahaishi | 2200-1900 | 2 | bone collagen | 2190-1905 | 10 | bone collagen | -7.6 | 0.4 | 8.2 | 0.9 | [[75](#_ENREF_75)] |
|  | Qijiaping | 1500-1250 | 2 | bone collagen | 1610-1130* | 42 | bone collagen | -8.9 | 1.1 | 9.8 | 0.9 | [[96](#_ENREF_96)] |
|  | Mogou | 1750-1100 | 8 | bone collagen | 1750-1100* | 85 | bone collagen | -14.4 | 1.7 | 9.3 | 1.2 | [[44](#_ENREF_44), [101](#_ENREF_101)] |
| **Yellow River Valleys, China** | Shimao | 2055-1645 | 4 | bone collagen | 2055-1645 | 4 | bone collagen | -8.4 | 0.1 | 6.9 | 0.9 | [[94](#_ENREF_94)] |
|  | Muzhuzhuliang | ca. 2000 | 1 | bone collagen | 1950-1780 | 8 | bone collagen | -8.2 | 1.5 | 8.8 | 0.6 | [[97](#_ENREF_97)] |
|  | Xunyi | 2395-1985 | 3 | bone collagen | 2395-1985 | 3 | bone collagen | -7.1 | 0.1 | 8.2 | 0.1 | [[94](#_ENREF_94)] |
|  | Qingliangsi | 2500-1500 |  |  |  | 14 | bone collagen | -7.9 | 0.6 | 8.8 | 1.0 | [[91](#_ENREF_91)] |
|  | Taosi | 2300-1900 | 1 | human bone | 2110-1685* | 12/7† | bone collagen | -6.3 | 1.1 | 8.9 | 1.3 | [[90](#_ENREF_90)] |
|  | Niedian | 2000-1000 |  |  |  | 60 | bone collagen | -7.1 | 0.3 | 10.5 | 0.7 | [[95](#_ENREF_95)] |
|  | Liuzhuang | 2000-1600 |  |  |  | 21 | bone collagen | -8.2 | 1.9 | 9.7 | 1.5 | [[93](#_ENREF_93)] |
|  | Yinxu | 1400-1100 | 32 | bone collagen | 1595-835* | 39/1† | bone collagen | -8.2 | 2.5 | 5.9 | - | [[88](#_ENREF_88)] |
|  | Xin`anzhuang | 1600-1046 |  |  |  | 39 | bone collagen | -9.0 | 0.7 | 10.0 | 0.7 | [[98](#_ENREF_98)] |
|  | Nancheng | 2100-1600 |  |  |  | 75 | bone collagen | -7.0 | 1.0 | 9.4 | 0.6 | [[100](#_ENREF_100)] |
|  | Xinglongwa Ш | 1500-1300 | 3 | bone collagen | 1510-1295 | 9 | bone collagen | -7.0 | 0.6 | 9.8 | 1.0 | [[40](#_ENREF_40)] |
|  | Nanzhai | 1800-1500 | 10 | bone collagen | 1895-1505* | 9/0† | bone collagen | -9.6 | 1.3 | - | - | [[88](#_ENREF_88)] |
|  | Yanshishangcheng | 1600-1400 | 3 | bone collagen | 1605-1290* | 3/0† | bone collagen | -7.6 | 0.8 | - | - | [[88](#_ENREF_88)] |
|  | Erlitou | 1750-1500 | 1 | bone collagen | 1880-1440* | 21/4† | bone collagen | -9.0 | 2.1 | 10.2 | 1.8 | [[90](#_ENREF_90)] |
|  | Wadian | 2200-1900 |  |  |  | 12 | bone collagen | -11.0 | 2.1 | 8.2 | 1.3 | [[99](#_ENREF_99)] |
|  | Xinzhai | 2000-1600 | 13 | bone collagen | 1955-1535* | 8 | bone collagen | -9.6 | 1.4 | 9.0 | 1.0 | [[89](#_ENREF_89)] |
|  | Qianzhangda | 1700-1100 |  |  |  | 6/9† | bone collagen | -8.2 | 0.9 | 10.1 | 1.2 | [[92](#_ENREF_92)] |

† = Sites where the first number represents the # of individual measured for δ^13^C and the second # represents the individuals measured for δ^15^N.

*= Carbon dating results which are recalibrated to calendar ages using CALIB REV 7.1.0 with the IntCal13 curve [[84](#_ENREF_84)].

Table S3. Modern climate information for Bronze Age sites from north China, Central Asia, and Europe.

| **Location** | **Site name** | **Climate** | | | | | |
| --- | --- | --- | --- | --- | --- | --- | --- |
|  |  | **Station*** | **Climate Type** | **Elevation (m)** | **Av. Temp. (℃)** | **Mean Precipitation (mm/yr)** | **Reference** |
| **Europe** | Olmo di Nogara | Verona | Humid subtropical climate | 68 | 13 | 819 | http://www.climatemps.com/ |
|  | Grotta Misa | Tuscania | Hot-summer Mediterranean climate | 176 | 15 | 683 | http://en.climate-data.org/ |
|  | Felcetone | Tuscania | Hot-summer Mediterranean climate | 176 | 15 | 683 | http://en.climate-data.org/ |
|  | Rymnio | Rymnio | Humid subtropical climate | 309 | 14 | 564 | http://en.climate-data.org/ |
|  | Glubokoe Ozero Ⅱ | Luhansk | Hot humid continental climate | 87 | 8 | 490 | http://en.climate-data.org/ |
| **South Kazakhstan** | Oi-Dzhailau Ⅶ | Almaty | Hot humid continental climate | 847 | 9 | 581 | http://www.climatemps.com/ |
|  | Kyzyl Bulak-Ι | Almaty | Hot humid continental climate | 847 | 9 | 581 | http://www.climatemps.com/ |
| **North Kazakhstan** | Novoilinovka | Lisakovsk | Warm humid continental climate | 123 | 3 | 315 | http://en.climate-data.org/ |
|  | Lisakovsk | Lisakovsk | Warm humid continental climate | 123 | 3 | 315 | http://en.climate-data.org/ |
|  | Bestamak | Lisakovsk | Warm humid continental climate | 123 | 3 | 315 | http://en.climate-data.org/ |
|  | Tegiszhol | Astana | Warm humid continental climate | 347 | 2 | 308 | http://en.climate-data.org/ |
|  | Dariya | Astana | Warm humid continental climate | 347 | 2 | 308 | http://en.climate-data.org/ |
|  | Tashik | Astana | Warm humid continental climate | 347 | 2 | 308 | http://en.climate-data.org/ |
|  | Aschisu | Astana | Warm humid continental climate | 347 | 2 | 308 | http://en.climate-data.org/ |
|  | Akimbek | Astana | Warm humid continental climate | 347 | 2 | 308 | http://en.climate-data.org/ |
|  | Kyzylkol | Astana | Warm humid continental climate | 347 | 2 | 308 | http://en.climate-data.org/ |
| **South Siberia, Russia** | Teleutsky Vzvoz-I | Barnaul | Warm humid continental climate | 185 | 2 | 423 | http://www.climatemps.com/ |
|  | Firsovo-XIV | Barnaul | Warm humid continental climate | 185 | 2 | 423 | http://www.climatemps.com/ |
|  | Karasuk sites | Minusinsk | Warm humid continental climate | 254 | 1 | 341 | http://www.climatemps.com/ |
|  | Andronovo sites | Minusinsk | Warm humid continental climate | 254 | 1 | 341 | http://www.climatemps.com/ |
|  | Okunevo sites | Minusinsk | Warm humid continental climate | 254 | 1 | 341 | http://www.climatemps.com/ |
| **Xinjiang, China** | Xiabandi | Kashi | Cold desert climates | 1291 | 12 | 65 | http://www.climatemps.com/ |
|  | Gumugou | Ruoqiang | Cold desert climates | 889 | 12 | 25 | http://www.climatemps.com/ |
|  | Yanghai | Shanshan | Cold desert climates | 934 | 12 | 34 | http://en.climate-data.org/ |
|  | Tianshanbeilu | Hami | Cold desert climates | 739 | 10 | 35 | http://www.climatemps.com/ |
| **Gansu Corridor, China** | Huoshaogou | Yumen | Cold desert climates | 1497 | 7 | 58 | http://en.climate-data.org/ |
|  | Ganguai | Jiuquan | Cold desert climates | 1478 | 7 | 88 | http://www.climatemps.com/ |
|  | Wuba | Zhangye | Cold desert climates | 1462 | 8 | 131 | http://en.climate-data.org/ |
|  | Mozuizi | Wuwei | Cold desert climates | 1535 | 8 | 165 | http://en.climate-data.org/ |
|  | Shangsunjia | Xining | Cold semi-arid climates | 2263 | 7 | 371 | http://en.climate-data.org |
|  | Sanheyi | Haidong | Cold semi-arid climates | 2118 | 7 | 370 | http://en.climate-data.org |
|  | Hupo | Haidong | Cold semi-arid climates | 2118 | 7 | 370 | http://en.climate-data.org |
|  | Lajigai | Haidong | Cold semi-arid climates | 2118 | 7 | 370 | http://en.climate-data.org |
|  | Xiahaishi | Lanzhou | Cold semi-arid climates | 1518 | 9 | 316 | http://www.climatemps.com/ |
|  | Qijiaping | Linxia | Warm humid continental climate | 1890 | 7 | 492 | http://en.climate-data.org/ |
|  | Mogou | Mogou | Subtropical highland oceanic climate | 2114 | 9 | 610 | http://en.climate-data.org/ |
| **Yellow River Valleys, China** | Shimao | Yulin | Cold semi-arid climates | 1158 | 9 | 411 | http://en.climate-data.org/ |
|  | Muzhuzhuliang | Yulin | Cold semi-arid climates | 1158 | 9 | 411 | http://en.climate-data.org/ |
|  | Xunyi | Xianyang | Cold semi-arid climates | 479 | 15 | 550 | http://en.climate-data.org/ |
|  | Qingliangsi | Yuncheng | Cold semi-arid climates | 370 | 14 | 523 | http://en.climate-data.org/ |
|  | Taosi | Xiangfen | Cold semi-arid climates | 434 | 13 | 514 | http://en.climate-data.org/ |
|  | Niedian | Jinzhong | Cold semi-arid climates | 795 | 10 | 432 | http://en.climate-data.org/ |
|  | Liuzhuang | Hebi | Cold semi-arid climates | 64 | 15 | 559 | http://en.climate-data.org/ |
|  | Yinxu | Anyang | Cold semi-arid climates | 64 | 15 | 563 | http://en.climate-data.org/ |
|  | Xin`anzhuang | Anyang | Cold semi-arid climates | 64 | 15 | 563 | http://en.climate-data.org/ |
|  | Nancheng | Handan | Cold semi-arid climates | 55 | 14 | 542 | http://en.climate-data.org/ |
|  | Xinglongwa Ⅲ | Chifeng | Cold semi-arid climates | 57 | 8 | 361 | http://en.climate-data.org/ |
|  | Nanzhai | Yichuan | Humid continental climate | 206 | 14 | 637 | http://en.climate-data.org/ |
|  | Yanshishangcheng | Yanshi | Humid subtropical climate | 124 | 14 | 616 | http://en.climate-data.org/ |
|  | Erlitou | Yanshi | Humid subtropical climate | 124 | 14 | 616 | http://en.climate-data.org/ |
|  | Wadian | Yuzhou | Humid subtropical climate | 114 | 14 | 702 | http://en.climate-data.org/ |
|  | Xinzhai | Xinmi | Humid continental climate | 309 | 14 | 680 | http://en.climate-data.org/ |
|  | Qianzhangda | Tengzhou | Humid continental climate | 57 | 14 | 716 | http://en.climate-data.org/ |

*Modern climate stations are selected according to distance to analyzed sites and the availability of reliable climate information.
